# Injuries, negative consequences, and risk behaviors among both injured and uninjured emergency department patients who report using alcohol and marijuana

**DOI:** 10.4103/0974-2700.44679

**Published:** 2009

**Authors:** Robert Woolard, Janette Baird, Michael J Mello, Christina Lee, Magda Harington, Ted Nirenberg, Bruce Becker, Lynn Stein, Richard Longabaugh

**Affiliations:** Department of Emergency Medicine, Paul Foster School of Medicine, Texas Tech University, El Paso, TX, USA; 1 Injury Prevention Center at Rhode Island Hospital, Department of Emergency Medicine, Warren Alpert School of Medicine, Brown University, Providence, RI, USA; 2 Brown University Center of Alcohol and Addictions, Providence, RI, USA

**Keywords:** Alcohol, brief intervention, injury prevention, marijuana

## Abstract

**Background:**

Brief intervention (BI) to reduce hazardous drinking and negative consequences such as injury has been effective when given in the emergency department (ED). The effectiveness and effect of BI has varied between injured and uninjured ED patients. This study compares injured and uninjured ED patients who admit to alcohol and marijuana use to determine their need and their readiness for BI.

**Patients and Methods:**

Participants volunteered to enter a randomized controlled trial of BI to reduce hazardous alcohol and marijuana use. Adult ED patients who had had alcohol in the last month and smoked marijuana in the last year were recruited. Those patients who were admitted to hospital, were under police custody, or were seeking treatment for substance use or psychiatric disorder were excluded. Research assistants interviewed participants using a validated questionnaire. Data were analyzed using SAS (version 9.1). Binominal tests of proportions, t-test analyses, and transformations were conducted as appropriate.

**Results:**

Injured (*n* = 249) and uninjured (*n* = 266) study participants reported very high, statistically equivalent (*P* > 0.05), rates of binge drinking (4–5 days/month), marijuana use (13 days/month), driving under the influence of marijuana or alcohol (>49% in the last 3 months), injury (>83% in the last year), and other negative consequences (>64% in the last 3 months) prior to their ED visit. These behaviors and the consequences demonstrate a need for change. Both injured and uninjured subjects were ready to change (>56%) and confident they could change (>91%) alcohol and marijuana use.

**Discussion:**

ED patients who admit to alcohol and marijuana use also use other hazardous substances and participate in high-risk behaviors. In both injured and uninjured patients who admit using alcohol and marijuana, the ED visit is an opportunity to deliver BI to reduce alcohol and marijuana use and associated risk behaviors and the subsequent injury and negative consequences. Given their risk behaviors and experience of negative consequences, members of both injured and uninjured groups have an equal need for BI. Fortunately, in both groups, a high number of members express motivation to change.

## INTRODUCTION

In the emergency department (ED) and trauma center, trials of brief intervention (BI) for injured patients have recruited varied populations and reported varied, but overall positive, results.[1] Mello demonstrated that BI delivered by telephone after an ED visit for injury reduced the frequency of driving under the influence.[2] D'Onofrio reported that BI delivered to injured or uninjured patients with hazardous drinking was effective in reducing alcohol consumption, though no more effective than scripted advice.[3] In a multicenter trial in 14 EDs, Bernstein showed that BI delivered to injured or uninjured hazardous drinkers reduced alcohol consumption.[4] Longabaugh reported that BI reduced alcohol-related injuries and other negative consequences of alcohol use for injured problem drinkers in the ED, without reducing alcohol consumption.[5] It is not clear which ED patient group—injured or uninjured—will benefit most from BI or which benefits will be brought about by BI: reduced alcohol consumption, reduced injury and negative consequences, or both.

In theory and practice, during a “teachable moment” patients are more receptive to counseling and more ready to change.[6,7] Thus, we could predict more readiness to change and more response to BI from patients when an alcohol-related injury brings them to the ED. It has been shown that BI implemented in the ED may be more effective in those patients who report greater readiness to change their behaviors.[8] However, in the Longabaugh study, in the treatment condition, patients receiving BI after an alcohol-related injury (causing their ED visit) responded with the same reductions in alcohol-related injuries and negative consequences as patients whose injuries were not alcohol related. With counseling (BI) both groups had reduced alcohol-related injuries and negative consequences at 12-months' follow-up compared to the standard care group.[5] This finding challenges the notion that an even more “teachable moment” is created when an ED patient has experienced an injury that is immediately associated with their alcohol misuse. It supports the notion that the ED visit is an opportunity for identification and intervention in patients who may benefit from BI. These patients are at risk for future injury and negative consequences, whether their ED visits are for alcohol-related injuries or not.

If the positive response of injured patients to BI is not dependent on alcohol involvement in their injury, BI might be equally effective at reducing injury and negative consequences among groups of uninjured and injured ED patients. The group of ED patients who have the greatest need for BI are those who have high alcohol use, indulge in risk behaviors, and thereby put themselves at risk of injury and other negative consequences. In Longabaugh's trial, the patients with highest alcohol use, most risk behaviors, and most injury were those who reported using both alcohol and marijuana. In that study, ED patients were recruited if they were injured problem drinkers. A large proportion (50%) reported using marijuana as well. Patients who used marijuana reported more binge drinking, more driving under the influence, more prior injuries, and more negative consequences. In both the intervention and control groups these patients also had worse outcomes than those who only used alcohol.[9]

Patients who use alcohol and marijuana might be at greater risk for negative consequences because they are heavier alcohol users and they are greater risk takers. For example, they reported more instances of driving under the influence of alcohol and more episodes of alcohol binge drinking.[9] They can also be expected to have conjoint use of alcohol and marijuana. A large proportion of ED patients who screen positive for alcohol may be using both alcohol and marijuana. This group participates more in risk behaviors that need modification. They are an easily identifiable group using two Yes/No questions. Since they are at higher risk and experience more negative consequences, they have more need for BI and could benefit more from it. A BI designed for this group should address both alcohol and marijuana use.

Consideration of these concepts, led to investigation of the following hypotheses:

1. Injured ED patients who use alcohol and marijuana are more likely to have a history of hazardous alcohol use, risk behaviors, and injuries and negative consequences than uninjured patients.
2. Injured patients who use alcohol and marijuana are more motivated to change risk behaviors than uninjured patients.

Rejection of these hypotheses would support providing BI to all ED patients with histories of alcohol and marijuana use, irrespective of whether they were injured or uninjured. This study compares baseline data collected on injured and uninjured ED patients who were recruited to participate in a randomized controlled trial to test the effectiveness of BI in reducing hazardous alcohol and marijuana use and the risk behaviors, injury, and negative consequences associated with their use.

## PATIENTS AND METHODS

### Participants

In a busy academic urban trauma center ED, patients were screened on a representative sample of selected day, evening, night, and weekend hours between November 2003 and October 2006. Patients, 18 years and older, who report alcohol use in the last month and marijuana use in the last year were invited to participate. Only English-speaking patients were included in the study. Patients were excluded if they were admitted to the hospital, were in treatment for substance use or psychiatric disorders, were in police custody, or were unable to consent. Participants were compensated $35.00 for completion of the assessment. The study and all procedures were approved by the hospital Institutional Review Board (IRB).

When research staff were present all patients (11,403) attending the ED were approached and, if willing, were screened. Of those screened, 1553 were eligible; 515 (33%) patients agreed to participate and completed the 40-min baseline assessment. After completing the baseline assessment, the participants entered into a randomized controlled trial to to receive or not receive a brief counseling intervention aimed at reducing injury among users of alcohol and marijuana.

Of the participants who completed the baseline assessment, 48% were injured. The injured and uninjured patients were demographically similar. The average age of the participants was 28 years; 68% were Caucasian and 17% were Hispanic or Latino. On average, participants had completed 12.4 years of education. There were significantly fewer members from the minority communities in the injured (27%) group than in the uninjured group (38%) and significantly more males in the injured (78%) group than in the uninjured group (57%).

### Measures

The Alcohol Use Disorders Inventory Test (AUDIT),[10] consisting of ten questions, was used for screening for hazardous or harmful drinking in the past 12 months. The sum of the AUDIT items (scores range from 0 to 40) was used in the analyses. The sum indicates the severity of alcohol abuse. A score greater than 7 indicates harmful and hazardous alcohol use and a need for referral for assessment and intervention. The AUDIT has an established internal consistency (alpha = 0.80) and can reliably identify participants with harmful and hazardous alcohol drinking histories.

Alcohol, Marijuana, and Drug Use (AMD) was developed for this study, modeled on time line follow back. It consists of seven questions related to the quantity and number of days of use of alcohol, marijuana, and other drugs on each of the preceding 30 days. This measure provides data on the days of use of alcohol at binge levels (i.e., four or more drinks on the one occasion for females and five or more for males). We used number of days of use in the analyses.

High Risk Behavior (HRB) Inventory consists of six questions adapted from the Center for Disease Control's Youth Risk Behavior Surveillance System (YRBSS).[11] The questions asked were items that measured the risk behaviors most often reported by study participants. These items provided data on the frequency of wearing seat belts (always, sometimes, rarely, never); any driving under the influence, i.e., after drinking, binge drinking, or after using marijuana (Yes/No and number of episodes); and any motor vehicle crash or fight (Yes/No and number of episodes) in the last 3 months. In the analyses we considered the responses as binary: Rarely/Never *vs* Always/Sometimes and Yes *vs* No.

Injury Behavior Checklist (IBC)[12] has questions on 17 categories of injuries occurring in the 12 months preceding the ED visit (not including the injury that brought them to the ED on the present occasion). This measure asks for the total number of injuries and the number related to consumption of alcohol and/or marijuana within 2 h of the injury in each of the 17 injury categories. The sum totals of all categories of injuries, injuries attributed to alcohol, and injuries attributed to marijuana use were used in the analyses.

Noteworthy Index of Problems (NIP; unpublished survey) has 19 questions about social, physical, and psychosocial negative consequences. Each question was asked twice: first with reference to consequences following alcohol use and, second, with reference to consequences following marijuana use during the last 3 months. Each question has five responses, scored from 0–4 (never, once or twice, sometimes, frequently, daily or almost daily). Each sum of the scores on questions concerning negative consequences associated with alcohol and marijuana was used in the analyses.

Contemplation for Change measures readiness to change drinking and marijuana use on two separate six-point Likert scales (1 = not ready for change; 2 = considering making some change sometime in the next few months; 3 = seriously considering making change; 4 = already making change; 5 = maintaining new changes; 6 = relapsed from change) Responses 3, 4, and 5 were considered to indicate ‘motivated’ or ‘ready to change.’

Confidence for Change measures confidence in ability to change alcohol and marijuana use on two separate four-point Likert scales (1 = not at all confident, 2 = somewhat confident, 3 = mostly confident, 4 = totally confident). Responses 3 and 4 were considered to indicate ‘confident in ability to change.’

### Data analysis

Statistical analyses were conducted using SAS (version 9.1). Analyses of data involving counts (HRB; Contemplation for Change, and Confidence for Change) were conducted using binomial test of proportions; t-test analyses were conducted on differences between groups on continuous data (AUDIT, AMD, NIP). Prior to data analyses the distribution of data was examined and due to non-normality a square root transformation was conducted on the variables obtained from the AMD and a log transformation was conducted on the two IBC sums. Results are presented with 95% confidence intervals and were considered to be significant at *P* <.05.

## RESULTS

### Substance use and risk behaviors

The mean AUDIT score for the injured group was 8.5 (SD = 6.6) and it was 8.2 (SD = 7.2) for the uninjured group (t(480) = 0.54; *P* = 0.60.) This indicates that, on average, participants in each group are using alcohol at similarly harmful and hazardous levels, above the score indicating a need for referral for assessment and intervention. As can be seen from Table 1 both injured and uninjured participants were similarly heavy users of substances, with 13 days of marijuana use, 8–10 days of alcohol use, and 1–2 days of other drug use each month. The only significant difference seen in substance use was that the injured group reported more days of alcohol use. To adjust for the effect of unequal distribution of gender and race (important demographic variables that are related to alcohol consumption) among the groups, we conducted a covariance analysis adjusting for the effects of gender and ethnicity. Once we adjusted for the covariates, the effects of injury *vs* non-injury were no longer significant for mean days of alcohol use [F(1, 437) = 2.69; *P* = 0.10]. Both groups reported equally high rates of alcohol-related risk behaviors such as binging and driving after binging on alcohol. Those who reported driving after too much to drink in the past 30 days averaged 3 such episodes. Many reported driving within 2 h of using marijuana in the past 3 months and individual drivers averaged 22 episodes. There were no significant differences between the groups in the report of lack of seat belt use or being involved in a physical fight or motor vehicle crash.

**Table 1:** Reported substance use and risk behaviors

Measure	Injured (I) *n* = 249	Uninjured (U) *n*= 266	Statistic
Alcohol, marijuana, and drug use (last 30 days)			
Mean days alcohol use	10 (95% CI: 8 to 12)	8 (95% CI: 7 to 9)	t(477) = 2.28; *P* = 0.02
Mean days marijuana use	13 (95% CI: 11 to 15)	13 (95% CI: 11 to 15)	t(477) = 0.22; *P* = 0.82
Mean days other drug use	2 (95% CI: 1 to 3)	1 (95% CI: 0.5 to 1.5)	t(477) = 1.12; *P* = 0.26
Mean days of binge alcohol	5 (95% CI: 4 to 6)	4 (95% CI: 3 to 5)	t(477) = 1.16; *P* = 0.23

High risk behaviors (last 3 months)			
Never or rarely use seatbelts	45%	56%	Z= −2.50; *P* = 0.01
Driving after too much to drink	19%	20%	Z = 10.30; *P* = 0.74
Driving within 2 h of marijuana	49%	50%	Z = −1.14; *P* = 0.26
Driving after alcohol binge	23%	23%	Z = −0.56; *P* = 0.58
Motor vehicle crash	12%	8%	Z = 1.58; *P* = 0.11
Fights	33%	26%	Z = 1.74; *P* = 0.08

### Injuries and negative consequences

Both groups reported similar high levels of injury and negative consequences. The injured group reported experiencing significantly more injuries in the past 12 months than the uninjured group (injured mean (M) = 12.3; uninjured M = 11.0; t(512) = 2.17; *P* = 0.03.) However, there were no differences between the two groups in the number of injuries ascribed to alcohol and/or marijuana use. In the 3 months prior to the ED visit there were no differences in the mean number of negative consequences (NIP) related to alcohol or marijuana use reported by both groups (NIP alcohol scale: injured M = 8.9, uninjured M = 9.3; t(489) = −0.33; *P* = 0.74: NIP marijuana scale: injured M = 4.7, uninjured M = 5.2; t(489) =–0.71; *P* = 0.48).

### Motivation and confidence for change

In both groups, similar proportions of participants were contemplating changing alcohol use (injured = 67% *vs* uninjured = 73%; Z = 1.33; *P* = 0.18), but a greater proportion of the uninjured group was contemplating changing marijuana use (injured = 53% *vs* uninjured = 66%; Z = 2.72; *P* = 0.01). Both injured (97%) and uninjured (97%) were confident they could decrease alcohol use (Z = −0.05; *P* = 0.96) and both injured (92%) and uninjured (91%) were confident they could decrease marijuana use (Z = −0.19; *P* = 0.86).

## DISCUSSION

In this study both injured and uninjured ED patients who volunteered to participate in a trial of BI had high rates of risk behaviors such as binge drinking (4–5 days in the last month) and driving impaired (at least once in the last 3 months for 49–50% of participants.) Most reported consequences of use in the last 3 months and injury related to use in the last year. Most were motivated to change their alcohol and marijuana use. Almost all were confident in their ability to change their use.

Injury is often the result of high-risk behaviors that include alcohol binge use and driving under the influence. To a lesser degree, other substances, particularly marijuana which is often used in combination with alcohol, contribute to injury. In this study, driving under the influence of marijuana in the last 3 months was reported by 49–50% of participants and driving after binge drinking was reported by 23%. These patients who report high-risk behaviors and have a heavy burden of negative consequences and injury need, and should benefit from, a focused BI designed to motivate them to change their alcohol and marijuana use and associated risk behaviors. This study shows that ED patients who admit to use of marijuana and alcohol, whether they are injured or not, are at similarly high risk of experiencing negative consequences (>64% had experienced negative consequences in the last 3 months) and injuries (>83% had experienced injury in the last year.) On average, members of the injured and uninjured groups experienced 11–12 injuries in the last year. Study participants were also motivated to change, with >56% being ready to change and >91% being confident they could change alcohol and marijuana use). These high levels of risk behaviors, negative consequences, and readiness to change demonstrate a need and desire for behavior change.

Patients using alcohol and marijuana can be expected to benefit from BI. Alcohol screening is now required at major trauma centers and is already underway in many EDs. Those screened for alcohol may also be using marijuana, and this possibility should be routinely addressed in the ED. Reduction of both alcohol and marijuana use, and the associated risk behaviors, are important targets for achieving good health outcomes in the many patients who use both substances. Studies should be conducted to test whether a BI designed to specifically to address alcohol and marijuana use will be successful in reducing use of both substances and the associated risk behaviors as well as negative consequences and injuries.

### Limitations

There were two demographic differences (gender and minority status) between the injured and uninjured groups. These differences in gender and minority status could be expected to cause differing levels of alcohol and marijuana use, risk behaviors, negative consequences, and injury. There were more males among the injured patients and this can be expected to account for increased substance use, risk behavior, negative consequences, and injury seen in that group. However, despite these demographic differences, we found only small (though statistically significant) differences between the injured and uninjured groups in days of alcohol use (10 *vs* 8), never or rarely using seat belts (45 *vs* 56%), total injuries (12 *vs* 11), and motivation to change marijuana use (53 *vs* 66%); there were no differences in the other measures. When an analysis of variance was conducted which included demographic differences, the difference between injured and uninjured with regard to days of alcohol use was entirely explained by the demographic differences. The demographic differences between injured and uninjured may be factors that influence response to BI, but these differences do not appear to greatly affect the high-risk profile, high burden of negative consequences, or high level of motivation found in both injured and uninjured groups.

A significant limitation of this study is the large number of eligible patients (67%) who did not give consent for inclusion in the study. Patients who agreed to participate underwent a 40-min baseline assessment followed by a 40-min counseling session in the ED and a second 40-min counseling session 1 week after the ED visit. They also agreed to two additional 40-min assessments at 3 months and 1 year. This demand on their time may have been one reason why many patients refused to participate. Also, it is likely that patients who did not consent did not feel they needed counseling or were not ready to change alcohol and marijuana behaviors. The study excluded those patients who were under treatment or specifically seeking treatment for alcohol or substance use. Since patients who volunteered to enter the study knew they could receive counseling, we speculate that their readiness for change was greater than that of those who did not consent.

Many ED patients admitted to alcohol and marijuana use but only some (33%) were willing to enter a research project involving counseling in the ED with follow-up. The challenge is to get more at-risk patients to participate. The large number of patients who did not consent for this study reveals the magnitude of this problem. The participants in this trial were those willing to undergo counseling in a research study. We submit that this is the population of interest since data from this group of study volunteers should be reflective of those ED patients who admit to alcohol and marijuana use and accept counseling for behavior change.

In this study, when the research staff was present in the ED, all patients not being admitted to hospital (with few exceptions) were approached and screened; 13.6% admitted to alcohol and marijuana use and 4.5% participated in the study, agreeing to counseling that involved an immediate session followed by a second session 1 week later. In an ED averaging 100 adult visits daily (less than in many busy EDs), screening would yield 31.5 referrals to counseling each week. This seems enough to justify a screening and intervention program for alcohol and marijuana, especially in similar busy, urban trauma center EDs.

## CONCLUSIONS

Many emergency physicians are aware of the link between alcohol and injury and many screen their injured patients for alcohol problems. In this study, we found that injured as well as uninjured ED patients who admit to using alcohol in the last month and marijuana in the last year are at high risk of injury and negative consequences. Most of these patients indicate that they are ready to, and confident that they can, change their behaviors. Emergency physicians should consider screening all ED patients for both alcohol and marijuana use. When patients admit use of both, they should be offered referral for assessment and intervention. Future studies should determine the benefit of a BI in the ED designed to reduce alcohol and marijuana use and associated risk behaviors among ED patients who use both substances and attending the ED for injury or illness.
